# Sensitivity Challenge of the Next-Generation Bolometric Double-Beta Decay Experiment

**DOI:** 10.34133/research.0569

**Published:** 2024-12-19

**Authors:** Long Ma, Huan-Zhong Huang, Yu-Gang Ma

**Affiliations:** ^1^ Key Laboratory of Nuclear Physics and Ion-Beam Application (MOE), Institute of Modern Physics, Fudan University, Shanghai 200433, China.; ^2^ Department of Physics and Astronomy, University of California, Los Angeles, CA, USA.

## Abstract

Cryogenic crystal bolometer plays a crucial role in searching for neutrinoless double-beta (0νββ) decay, which is a rare process that could determine the Majorana nature of neutrinos. The flagship bolometer experiment—CUORE (Cryogenic Underground Observatory for Rare Events)—operating at the Gran Sasso underground laboratory [Laboratori Nazionali del Gran Sasso (LNGS)] as the world’s first ton-scale bolometric detector has achieved great success and well demonstrated advantages of the bolometric technology for the 0νββ study. The proposed upgrade of CUORE—the CUPID project—aims to achieve higher sensitivity with orders of magnitude background reduction by utilizing scintillating crystals and dual readout technology to exclude most of the background events dominated by alpha particles. Although CUPID has outstanding advantages over CUORE, further increasing the detection capability to fully explore the effective neutrino mass region for the inverted neutrino mass hierarchy and possibly to discover Majorana neutrinos remains a technical challenge ahead. In this prospective, we discuss strategies toward future technology development to further enhance the experimental sensitivity.

The observation of neutrinoless double-beta (0νββ) decay would have great impact on fundamental particle physics as it provides direct proof of lepton number violation, demonstrates the Majorana nature of neutrino, and helps to explain the matter–antimatter asymmetry in the universe. Currently, several 0νββ experiments are actively searching for signals indicative of the 0νββ decay [1–9]. Of course, as an important step for the searches of Majorana neutrinos with 0νββ decay, precise measurement of 2-neutrino double-beta decay (2νββ) half-life is also important [10–14]. Among all the 0νββ efforts, bolometers, also known as low-temperature calorimeters, show particular advantages as a long-term established technology employed in rare event search. These solid-state detectors operate at temperatures approaching absolute zero, enabling the detection of rare processes through heat (phonon) sensors, which register the temperature increase resulting from energy released within the detector target [15]. The bolometer technology offers several advantages including high energy resolution, high detection efficiency, and flexibility in isotope selection.

As the world’s largest bolometer experiment, CUORE (Cryogenic Underground Observatory for Rare Events) employs an array of 988 cubic TeO_2_ crystals, a total mass of 742 kg, to measure potential 0νββ signals from the ^130^Te isotopes (Figure) [1]. The recent findings of CUORE based on the 1 ton-year exposure data mark a substantial advancement in the bolometric 0νββ study. Operating at a temperature close to 10 mK and protected by dedicated means against radioactivity, CUORE pushed the detection sensitivity to a world’s competitive level with a mean background rate of 0.0149 counts/(keV kg year) at the 0νββ *Q* value (2527 keV) of ^130^Te. Although no evidence of 0νββ decay is observed in the data, a world-leading limit of 2.2 × 10^25^ years at a 90% credibility interval was set for the 0νββ half-life of ^130^Te. This result corresponds to an effective Majorana neutrino mass m_ββ_ < 90 to 305 meV, accounting for uncertainties in nuclear matrix element calculations, representing one of the most stringent constraints up to now in 0νββ experiments based on different isotopes [1]. Further background identification and suppression are necessary to advance the bolometer detection technology for 0νββ physics [13].

**Figure.**
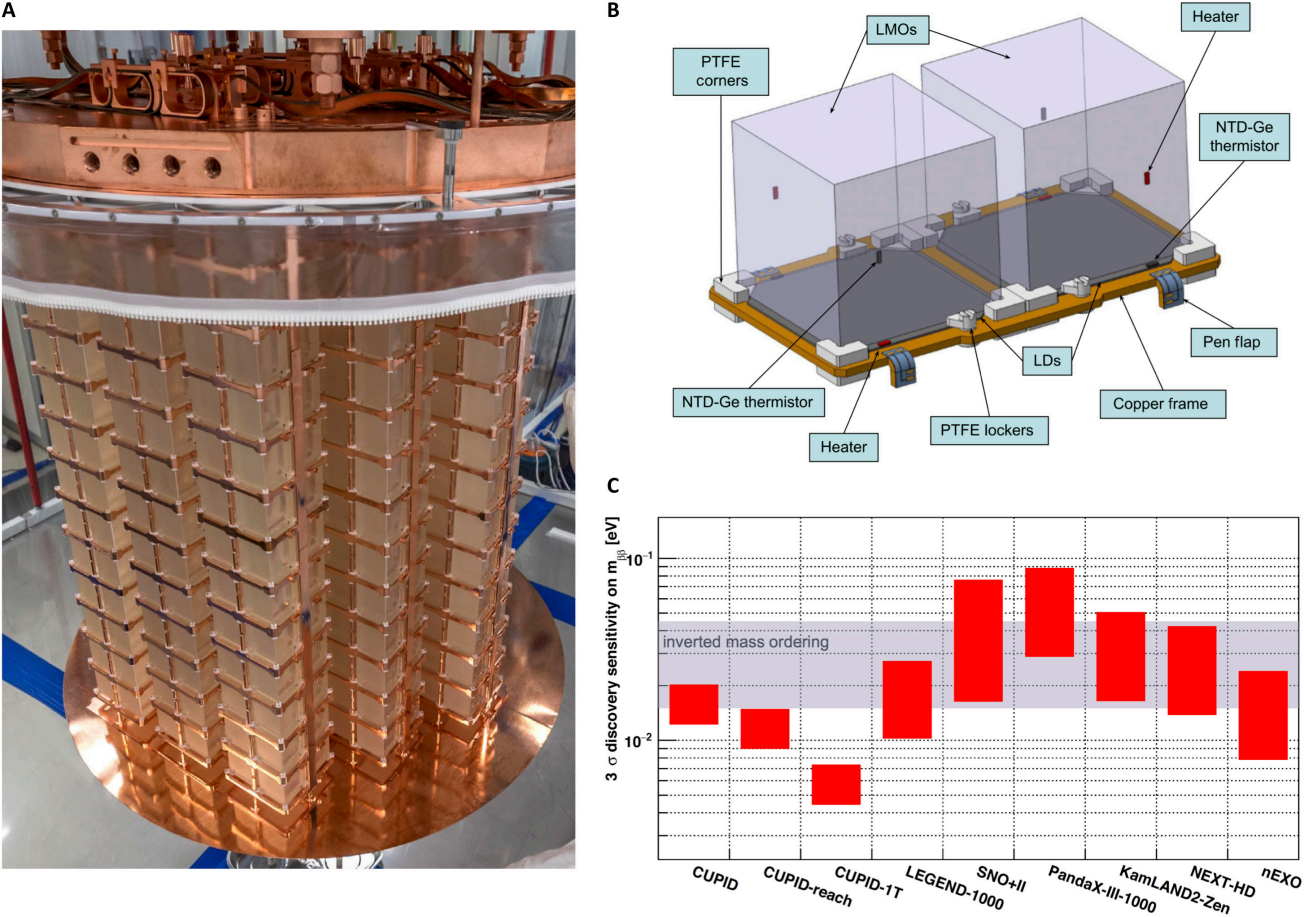
(A) The CUORE detector after installation. An array of 988 TeO_2_ crystals is hosted in the cryostat [1]. (B) Schematic plot of a single CUPID module consists of cubic LMO crystals and light detectors. The detector components including copper frame and poly-tetrafluoroethylene (PTFE) elements are labeled [16]. (C) Sensitivity projection for the next-generation ton-scale experiments. The gray band shows the neutrino inverted mass ordering region [18].

To further increase the experimental sensitivity, the new-generation bolometric 0νββ experiment referred to as CUPID (CUORE Upgrade with Particle IDentification) is proposed based on scintillating bolometer technology and valuable expertise from CUORE. The scintillating bolometers utilize inorganic crystal scintillators, detecting simultaneously heat (phonon) and scintillation signals, which is essential for background identification and rejection, dramatically extending detector performance compared to the conventional cryogenic bolometer. The development of the lithium molybdate [Li_2_MoO_4_ (LMO)] scintillating bolometer in recent years marks a significant step forward for the future CUPID double-beta decay experiments [16]. Efforts have been devoted to validate the technology and perform small-scale demonstration experiments [14,17]. Innovative setups with natural or ^100^Mo-enriched LMO crystals and germanium (Ge) light sensor have been constructed and tested (Figure) [16]. Technical validation runs allowed comprehensive assessments of the radioactive contamination levels in the LMO crystals, providing valuable insights into the background contributions to the 0νββ energy region of interest (ROI). The performance of the light-heat dual readout was evaluated, and excellent α background separation (from β/γ events) has been achieved.

Despite the technical advantages of CUPID, achieving a significant increase in sensitivity fulfilling the complete coverage of the inverted ordering region of the neutrino effective mass still presents a major challenge (Figure) [18]. Background level has always been a major challenge to the experimental sensitivity. In the conservative baseline scenario of CUPID, a background index (BI) of 10^−4^ counts/(keV kg year) in ROI is required. Moreover, BI < 10^−5^ counts/(keV kg year) is required for the future ton-scale experiment CUPID-1T using ^100^Mo-enriched LMO. Developing strategies for further background mitigation is an urgent priority. Pilot experiments including CUPID-0 and CUPID-Mo indicate that using high Q_ββ_ isotopes and dual readout techniques, one can effectively suppress most of the backgrounds especially α events originating from environmental and material radioactivity near the *Q* value. But, as these backgrounds become less dominant, the influence of 2νββ and pile-up events becomes increasingly prominent. Therefore, improving the time resolution of detector and further optimizing the design such as crystal size are directions necessary for further exploration.

In addition, cosmogenic background contribution needs to be given special attention. Normally, operating detectors in deep underground laboratory with substantial rock overburden effectively minimize cosmic radiation. But, to achieve a stringent background level aligning with the requirements of CUPID, anti-coincidence setups like muon veto are necessary. Moreover, underground detector material production is an effective low-background approach [19,20]. Besides, efforts should be devoted to the optimization of the detector structure to achieve an improved light collection relative to current performance. Through continuous optimization, one could achieve an optimal configuration essential for the future large-scale experiments.

Currently, several countries around the world are actively engaged in the development of the CUPID-like bolometer technology. Notably, as part of the international CUPID consortium, the Chinese team is focusing its efforts on the research and development of CUPID technology at China Jinping Underground Laboratory (CJPL). CJPL is currently the world’s deepest underground laboratory in terms of the rock overburden, with world-leading low cosmogenic background environment. Leveraging its advanced crystal production capabilities, the Chinese team plans to conduct comparative studies of the NTD-Ge (Neutron Transmutation Doped Germanium) sensor and TES (Transition Edge Sensor)-based readout employed by CUPID light detectors. Additionally, collaborative efforts will also be devoted to optimize detector designs and acquire the necessary expertise to build and operate a future ton-scale bolometer experiment at CJPL.

In summary, significant efforts have been invested in the development of the CUPID technology and the operation of demonstration experiments for the 0νββ study. The CUPID technology for LMO bolometers with phonon-scintillation dual readout, using Ge light detectors, has been systematically validated by pilot experiments, well demonstrating the technological competitiveness in background mitigation. In the future, to achieve orders-of-magnitude improvement in sensitivity of the next-generation 0νββ experiments, additional background mitigation strategies need to be implemented. With continuous breakthroughs in low-background detection technology, bolometric experiments are expected to play critical roles in the search for rare physical events beyond the standard model physics.
